# Linear interactions between intraocular, intracranial pressure, and retinal vascular pulse amplitude in the fourier domain

**DOI:** 10.1371/journal.pone.0270557

**Published:** 2022-06-28

**Authors:** Anmar Abdul-Rahman, William Morgan, Ying Jo Khoo, Christopher Lind, Allan Kermode, William Carroll, Dao-Yi Yu

**Affiliations:** 1 Department of Ophthalmology, Counties Manukau District Health Board, Auckland, New Zealand; 2 Centre for Ophthalmology and Visual Science, The University of Western Australia, Perth, Australia; 3 Lions Eye Institute, University of Western Australia, Perth, Australia; 4 Royal Perth Hospital, Perth, Australia; 5 Neurosurgical Service of Western Australia, Sir Charles Gairdner Hospital, Nedlands, Western Australia, Australia; 6 Department of Surgery, University of Western Australia, Crawley, Western Australia, Australia; 7 Centre for Neuromuscular and Neurological Disorders, Perron Institute for Neurological and Translational Science, Sir Charles Gairdner Hospital Department of Neurology and Clinical Neurophysiology, Nedlands, Western Australia, Australia; 8 Institute for Immunology and Infectious Disease, Murdoch University Faculty of Health Sciences, Murdoch, Western Australia, Australia; Icahn School of Medicine at Mount Sinai, UNITED STATES

## Abstract

**Purpose:**

To compare the retinal vascular pulsatile characteristics in subjects with normal (ICP_n_) and high (ICP_h_) intracranial pressure and quantify the interactions between intraocular pressure, intracranial pressure, and retinal vascular pulse amplitude in the Fourier domain.

**Materials and methods:**

Twenty-one subjects were examined using modified photoplethysmography with simultaneous ophthalmodynamometry. A harmonic regression model was fitted to each pixel in the time-series, and used to quantify the retinal vascular pulse wave parameters including the harmonic regression wave amplitude (HRW_a_). The pulse wave attenuation was measured under different ranges of induced intraocular pressure (IOP_i_), as a function of distance along the vessel (V_Dist_). Intracranial pressure (ICP) was measured using lumbar puncture. A linear mixed-effects model was used to estimate the correlations between the Yeo-Johnson transformed harmonic regression wave amplitude (HRW_a-YJt_) with the predictors (IOP_i_, V_Dist_ and ICP). A comparison of the model coefficients was done by calculating the weighted Beta (*β*_*x*_) coefficients.

**Results:**

The median HRW_a_ in the ICP_n_ group was higher in the retinal veins (4.563, interquartile range (IQR) = 3.656) compared to the retinal arteries (3.475, IQR = 2.458), p<0.0001. In contrast, the ICP_h_ group demonstrated a reduction in the median venous HRW_a_ (3.655, IQR = 3.223) and an elevation in the median arterial HRW_a_ (3.616, IQR = 2.715), p<0.0001. Interactions of the pulsation amplitude with ICP showed a significant disordinal interaction and the loss of a main effect of the Fourier sine coefficient (b_n1_) in the ICP_h_ group, suggesting that this coefficient reflects the retinal vascular response to ICP wave. The linear mixed-effects model (LME) showed the decay in the venous (HRW_a-YJt_) was almost twice that in the retinal arteries (−0.067±0.002 compared to −0.028±0.0021 respectively, p<0.00001). The overall interaction models had a total explanatory power of (conditional R^2^) 38.7%, and 42% of which the fixed effects explained 8.8%, and 5.8% of the variance (marginal R^2^) for the venous and arterial models respectively. A comparison of the damping effect of V_Dist_ and ICP showed that ICP had less influence on pulse decay than distance in the retinal arteries (*β*_*ICP*_ = -0.21, se = ±0.017 compared to $\beta_{V_{\text{Dist}}}=-0.26$, se = ±0.019), whereas the mean value was equal for the retinal veins (venous $\beta_{V_{\text{Dist}}}=-0.42$, se = ±0.015, *β*_*ICP*_ = -0.42, se = ±0.019).

**Conclusion:**

The retinal vascular pulsation characteristics in the ICP_h_ group showed high retinal arterial and low venous pulsation amplitudes. Interactions between retinal vascular pulsation amplitude and ICP suggest that the Fourier sine coefficient b_n1_ reflects the retinal vascular response to the ICP wave. Although a matrix of regression lines showed high linear characteristics, the low model explanatory power precludes its use as a predictor of ICP. These results may guide future predictive modelling in non-invasive estimation of ICP using modified photoplethysmography.

## Introduction

Changes in retinal hemodynamics are a consequence of a complex interaction between intraocular pressure (IOP), intracranial pressure (ICP), and systemic blood pressure [1, 2]. The balance of cerebrospinal fluid, blood supply, and cerebral parenchymal volume is required for ICP homeostasis [3]. Although the outflow of cerebrospinal fluid into the venous circulation accommodates a moderate increase in ICP, once the buffering mechanisms are decompensated, ICP rises to the level of intravascular pressure in the cerebral arterioles [4], consequently changes occur in the retinal vasculature. Increased retinal venous pressure causes venous distension, which subsequently reduces venous compliance [5–7]. Several human and animal studies confirmed the strong linear relationship between retinal venous hemodynamic parameters and ICP. Using ophthalmodynamometry to assess the correlation between venous opening pressure and ICP, Firsching et al. found a linear correlation (R) of 0.983 (p<0.001), Motshcmann et al. similarly reported a correlation of 0.968 [8]. Querfurth et al. measured the correlation of venous opening pressure (R = 0.87) in addition to the inverse correlation of the arterial pulsatility indices for both the ophthalmic and central retinal arteries (R = 0.66) they described an empiric index by combining both venous and arterial parameters (venous opening pressure/Gosling Pulsatility Index [GPI]) that was more strongly correlated with absolute ICP than either parameter alone (R = 0.95, p <0.005) [9]. Similarly, Hayreh et al., in a primate study, reported ophthalmic venous pressure to be linearly correlated with ICP (R^2^ 0.8–1, p = 0.01) when measured using direct cannulation [5]. The influence of ICP on the retinal arterial system is more controversial. In 1927 Baurman provided the earliest description of retinal hemodynamic changes with acute elevation of the ICP; using ophthalmodynamometry he reported a linear relationship between ICP and retinal venous pressure [10]. Using the same methodology Berens and co-workers found a raised pressure in the central retinal artery independent of systemic blood pressure [11]. In contrast in a primate model, Hayreh et al. demonstrated a high positive correlation between the systemic blood pressure and ophthalmic artery pressure, they reported systolic ophthalmic artery pressure was 71% of the systolic blood pressure and the diastolic ophthalmic artery pressure 87% of the diastolic systemic pressure, the correlation between the diastolic ophthalmic and systemic blood pressures was maintained on raising ICP to 35mmHg. At higher ICPs, and the systolic ophthalmic artery pressure dropped to 47% of the systolic blood pressure. It was thought that this was due to a “cuffing effect” of the raised ICP on the intracranial portion of the ophthalmic artery, which did not interfere with the diastolic ophthalmic artery pressure [5]. It is likely that a complex interaction between IOP, local anatomical factors, retinal hemodynamics, ICP, retrolaminar tissue pressure, and systemic blood pressure influence this predictive accuracy [12]. This complex interaction of factors is traditionally summarised by the translaminar pressure gradient and difference. In these assumptions, cerebrospinal fluid pressure is considered as an opposing force to intraocular pressure at the lamina cribrosa [13–16]. Here, venous pulsations are thought to cease when ICP exceeds central venous pressure. Based on this assumption, several investigators have attempted to define this critical ICP threshold value, Walsh et al. [17]. showed that the mean cerebrospinal fluid pressure for obliterating pulsations was 200±25 mm Water. Similarly, Kahn and Cherry [18], Levin [19] and Rosenberg [20] showed ICP must be less than 190–195 mm Water when pulsations are present [21]. Yet paradoxically these reduced models and simplified assumptions have not yielded an adequate predictive power when ICP prediction is concerned [3, 22, 23]. The paradox may be due to unaccounted-for non-linear dynamics or inter and intra-individual variability of the predictors, which may further limit the practical applicability of these reduced models.

Uncertainty principles refer to a meta-theorem in Fourier analysis. They are expressed as inequality equations and convey the general concept that a non-zero function and its Fourier decomposition cannot be localized to arbitrary precision [24]. Of the uncertainty principles, the Heisenberg-Gabor uncertainty principle defines the limit of the resolution of a signal in both time and frequency. Described in 1946 in Gabor’s work on the theory of communication, it states that information cannot be localized simultaneously in both time and frequency [25]. Hence, the principle asserts that there exists a tradeoff between temporal (Δt) and spectral (Δf) resolution [26]. This is encapsulated by the mathematical identity:

$$\begin{array}{c} \Delta f\cdot \Delta t\geq C \end{array}$$

Where (C) is a constant with a value dependent on the methodology employed in frequency measurement. Here, for the inequality to remain true the reduction in temporal resolution demands an increase in frequency resolution. Additional to the aforementioned unique resolution property and hence information content, frequency domain analysis has the advantage of de-noising the decomposed signal, a characteristic that is favorable in artifact-prone ophthalmic imaging. Consequently, hemodynamic parameters can exist as a distinct property of the frequency domain, such as input impedance (defined as the ratio of the pressure to flow harmonics [27]), dispersion (defined as wave velocity dependence on harmonic frequency [28]), and harmonic amplitude attenuation (defined as the decay in harmonic amplitude as a function of distance [29]). Modified Photoplethysmography is a novel non-invasive technique of measurement of the retinal vascular pulse amplitude and timing characteristics in the Fourier domain. A plethysmography wave has non-periodic and periodic components [30]. Therefore, the harmonic regression model used to analyze the retinal vascular pulse wave consists of a linear spline to model the non-periodic component and an autoregressive error component used to represent the trend in the time series. The periodic component is represented by a Fourier series composed of the first two harmonics [31]. Akaike Information Criterion is used to determine the maximum extractable frequencies from the images. Generally, the number of extractable harmonics is dependent on the signal-to-noise ratio. Theoretical studies on vascular pressure-flow waveforms indicate that there are a larger number of extractable harmonics, Krovetz et al. [32]. analyzed the harmonics of intracardiac and arterial pressure waves, and they reported that 3–10 harmonics were required to reproduce the pressure waveforms, however, differential pressures and derivatives, appeared to contain six-fold the number of higher-frequency harmonics [32]. From other studies, the first two harmonics account for approximately 85% of the pulsatile portions of pressure and flow in large arteries, which may indicate that lower-order harmonics are sufficient to represent these waveforms [33]. Practically, there is a fundamental limit to the number of harmonics that can be extracted. Beyond the tenth harmonic component, the harmonic magnitudes of the aortic pressure pulse and aortic flow pulse are negligible. This limit arises from sampling factors and signal noise, as to accurately reconstruct the original waveform, the Nyquist criterion requires a sampling frequency of at least twice the highest-frequency content. As a result, frequency-domain methods rely on identifying a suitable frequency band or harmonic range that is less susceptible to noise [27, 34–36]. Although general principles around the Nyquist limit apply to harmonic pulse wave analysis, comparisons of the extractable harmonics in these studies with our work are not possible due to differences in the measurement parameter, methodology, and the vessel characteristics.

In this study, we quantified the correlation between the linear terms of pulse amplitude in the frequency domain for both the retinal venous and the arterial systems at different distances along the vessels and under a range of induced IOP values; generated by ophthalmodynamometry in patients with normal and high ICP. In order to ascertain the variance captured by a linear mixed effects model and further our understanding of the limit of linear models in the prediction of ICP non-invasively. Hence, the parameters of linear models with and without interactions between the predictors are estimated. Here, we make no assumptions regarding the physiology and physics of collapsible vessel behavior at pressure discontinuities.

## Materials and methods

### Study recruitment

**Fig 1:**
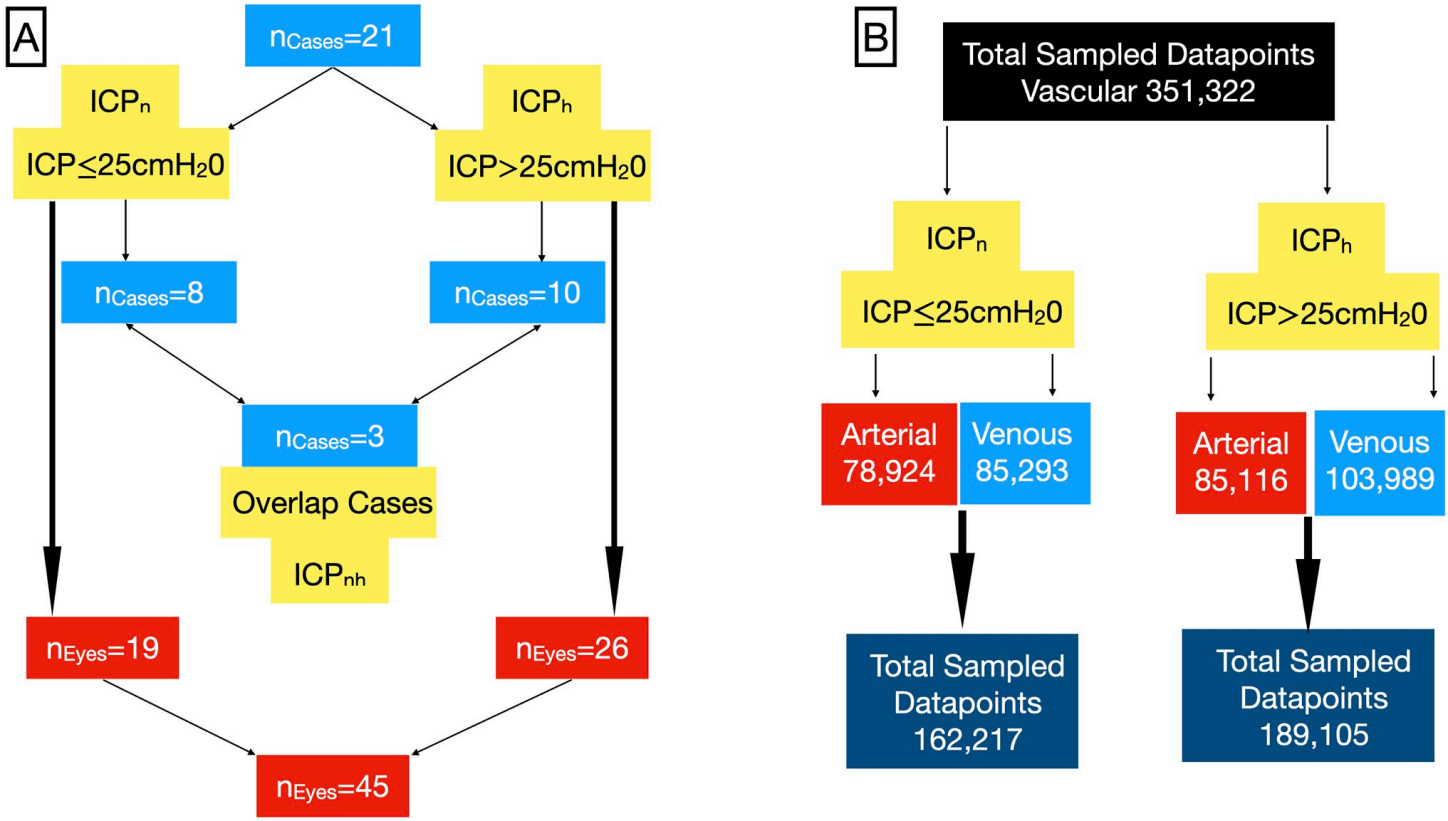
Classification of the study population and image data points. A) A total of 21 subjects were classified into two groups dependent on the intracranial pressure (ICP). An ICP of 25 cm water was the normal upper limit. B) A total of 351,322 data points were sampled from the images.

Participants were referred to the Lions Eye Institute over five years (2015–2020) from the Neurology and Neurosurgery Departments and were to undergo lumbar puncture for suspicion of idiopathic intracranial hypertension. Gender is a well recognised risk factor in this disorder with over 90% of patients affected with idiopathic intracranial hypertension are female [37]. This explains the gender bias in our study. Modified photoplethysmography, a form of ophthalmodynamometry, was performed on all subjects. This consists of a force transducer surrounding a central contact lens, which allows imaging of the optic disc under a dynamic range of induced intraocular pressures (IOP_i_). Written consent was obtained from each of the participants. Study approval was obtained from the University of Western Australia Human Ethics Committee adhering to the tenets of the Declaration of Helsinki. Participants were required to have clear ocular media. Exclusion criteria included any previous history of retinal or optic nerve pathology and optical media opacity. There were a total of 21 patients in the study group, an ICP of 25 cm water was considered the upper normal limit [38]. Ten cases (20 eyes) were in the high intracranial pressure group (ICP_h_>25cm water) and 8 cases (13 eyes) in the normal intracranial pressure group (ICP_n_≤25cm water). Three cases (6 eyes) overlapped between the two groups as a result of interchanging between the ICP_n_ to the ICP_h_ groups. Therefore there were a total of 19 eyes in the ICP_n_ group and 26 eyes in the ICP_h_ group, giving a total of 45 eyes in the study as three eyes were excluded from the analysis due to poor image quality (Fig 1). A total of 351,322 data points were sampled from the images of the study group: 162,217 arterial and 189,105 venous data points (Fig 1).

### Image acquisition

All participants underwent visual acuity and slit-lamp examination. Baseline intraocular pressure was measured with Goldmann contact tonometry. Photoplethysmography was performed using the Meditron ophthalmodynamometer (Meditron GmbH, Poststrasse, Völklingen, Germany). This device consists of a sensor ring, which measures the force surrounding a central Goldmann three-mirror contact lens. The optic nerve head was continuously imaged bio-microscopically during the examination through the ophthalmodynamometer central contact lens. The examination was repeated approximately 10 times for each subject to attain a range of ophthalmodynamometry values for each eye, allowing a video segment to span at least five consecutive cardiac cycles. Examination duration varied depending on the number and length of the video recordings. A maximum of 2–3 minutes of light exposure per eye was estimated for the duration of the test. Slit-lamp light intensity was set at the minimum setting allowed for imaging to improve subject comfort and compliance with the test. Although the effects of ocular warming on the retinal vascular pulse wave is a theoretical possibility, in our study the effect is likely to be negligible due to the brief duration of exposure and the lower light intensities compared to those reported in studies of the effect of temperature on retinal blood flow [39, 40].

Videos showing excessive motion artifact, reflection from the optical media, or decentration of the optic nerve in the image sequence for less than three consecutive cardiac cycles were rejected from the analysis. The ophthalmodynamometric force (ODF) displayed as Meditron units (mu) was converted to induced intraocular pressure (IOP_i_) estimated from the baseline intraocular pressure (IOP_b_) using the formula described in previous work [41]:

$$\begin{array}{c} IOP_{i}=0.89\cdot ODF+IOP_{b} \end{array}$$

An imaging slit-lamp (Carl Zeiss, Germany) with a digital camera (Canon 5D Mark III, Japan) was used to record multiple video sequences of at least three cardiac cycles in length, each at a rate of 25 frames/second. A pulse oximeter (Nellcor N65, Covidien, Mansfield, MA) was applied to the right index finger; the audio signal from the pulse oximeter, captured with the video sequence of the optic nerve allowed synchronization of the retinal vascular pulse with the cardiac cycle as image analysis required timing of the retinal vascular pulse based on fractions of the cardiac cycle. A Single high-quality length video spanning three-cardiac cycles was extracted from each recording session.

### Image analysis

Adobe Photoshop CS6 was in the image processing step, individual image frames were extracted from each video sequence and saved as Tagged Image File Format (TIFF) files. Each of these images was cropped to an array of pixels. All images from three cardiac cycles were analyzed in R statistical package using custom software [42]. Each data point was represented by the mean of the green channel intensity at time measured as a fraction of the cardiac cycle, rather than in seconds. The periodic trend component was modeled as a Fourier series expansion:

$$\begin{array}{c} F\left({f(t)}_{p}\right)=a_{0}+\sum_{n=1}^{\infty }a_{n}\cdot cos\left(n\pi t\right)+b_{n}\cdot sin\left(n\pi t\right) \end{array}$$

f(t)_p_ = The periodic component of the time series.

a_0_ = Coefficient representing the mean of f(t)_p_.

a_n_ = Coefficient of the cosine function of f(t)_p_.

b_n_ = Coefficient of the sine function of f(t)_p_.

n = Integer 0,1,2… etc representing the harmonic component.

Higher harmonic frequency model comparisons were conducted using Akaike Information Criterion. In most eyes the Akaike Information Criterion preferred models with first and second-order frequencies, therefore the final analysis was limited to the first and second harmonics (n1,2). Image analysis and segmentation are detailed in our previous work [29, 43].

### Statistical analysis

A harmonic regression model was fitted to each pixel in the time-series, and used to quantify the retinal vascular pulse wave parameters including the harmonic regression wave amplitude (HRW_a_). The model includes a Fourier series representation using the first and second harmonics, linear spline non-periodic component, and a first-order autoregressive error component. The model was applied to the retinal arteries and veins separately [29, 31, 44]. The distribution of the HRW_a_ and the majority of the Fourier coefficients were non-normal, therefore the median was used to measure central tendency, and dispersion was estimated using interquartile range (IQR), and the range was also computed. Hypothesis tests were conducted for both the non-normalized and normalized data using the Wilcoxon test with Bonferroni correction, and multivariate analysis of variance (MANOVA) respectively. Multifactoral Homogeneity of variance was tested using the Levene test.

Yeo-Johnson transformation (YJ_t_) was used to normalise the harmonic regression waveform amplitude (HRW_a_), the cosine (a_n1,2_) and the sine (b_n1,2_) coefficients of the first and second Fourier harmonics. The justification of this approach was evaluated by the transformation-estimating functions in the bestNormalize package in R [45], which uses the estimated normality statistics (Pearson P / df) statistic in recommending a transformation method and the required parameters (Table 1). The lower the normality statistic, the more favorable is the approximation to a normal distribution. This transformation is a suitable approach for negative values [46, 47]. These transformations are defined by:

$$\begin{array}{c} \psi \left(\lambda ,y\right)=\{\begin{array}{cc} \frac{{\left(y+1\right)}^{\lambda }}{\lambda } & if\;\lambda \neq 0,y\geq 0\\ log(y+1) & if\;\lambda =0,y\geq 0\\ \frac{{\left(-[y+1]\right)}^{2-\lambda }-1}{2-\lambda } & if\;\lambda \neq 2,y<0\\ -log(-y+1) & if\;\lambda =2,y<0 \end{array} \end{array}$$

**Table 1:** Estimated normality statistics.

	Non-Transformed	Yeo-Johnson Transformed	λ
**Artery**			
**HRW_a_**	35.4325	6.5197	-0.0635
**a_n1_**	2.6041	1.9499	0.9406
**b_n1_**	12.5317	12.7153	0.9747
**a_n2_**	5.6896	5.7172	0.8976
**b_n2_**	5.9913	5.8878	1.0475
**Vein**			
**HRW_a_**	63.0409	7.5351	-0.1684
**a_n1_**	21.6354	5.9935	0.7561
**b_n1_**	23.2509	23.0105	1.0128
**a_n2_**	11.1054	9.856	1.0871
**b_n2_**	12.5743	12.338	1.0561

Estimated normality statistics (Pearson P/df) comparing non-transformed with Yeo-Johnson Power transformed parameters of the Fourier series. The lower the normality statistic the more favourable approximation to a normal distribution. Lambda (λ) is the critical parameter for the Yeo-Johnson transformation.

If the data point is strictly positive, then the YJ_t_ is the same as the Box-Cox power transformation of (y+1). If strictly negative, then the YJ_t_ is the Box-Cox power transformation of (-y+1), but with power 2-λ. With both negative and positive values, the transformation is a mixture of these two methods [46]. The Estimated Normality Statistics (Pearson P / degrees of freedom) were used, this showed favorable transformation criteria for YJ_t_ data when compared against both non-transformed and Logarithmic transformation with translation (Table 1).

Interaction plots were created to determine the Fourier coefficients influenced by increased ICP. The regression equations consisted of a five-level hierarchical linear mixed-effects model, with and without interaction effects was used to analyze the correlations of both the HRW_a_ and individual trigonometric harmonic coefficients [cosine (a_n1,2_), sine (b_n1,2_)] of the first and second harmonics with the predictors, ICP, the distance along the vessel (V_dist_) and induced intraocular pressure (IOP_i_). Linear mixed effect model goodness fit statistics were assessed using conditional R^2^, which describes the proportion of variance explained by all factors in the model including the predictors, ICP, V_dist_ and IOP_i_ and random factors (subject, age, gender, laterality “right/left”, hemiretinal location “superior/inferior”), whereas marginal R^2^ describes the proportion of variance explained by the predictors ICP, V_dist_, and IOP_i_ alone [48]. Beta coefficients were computed to compare effect size in the multivariate regression models.

## Results

### Study population

The age of the population demonstrated a bimodal distribution with a mean of 30 years (range 17–47 years). There were 20 (95.2%) females and 1 (4.8%) male. Whereas in the ICP_n_ group the median was 18.50 cm water (range 9.50–24, IQR = 5.5), the corresponding values in the ICP_h_ group were 31cm water (range 25.50–68, IQR = 11).

### Harmonic regression waveform amplitude and Fourier coefficients

**Fig 2:**
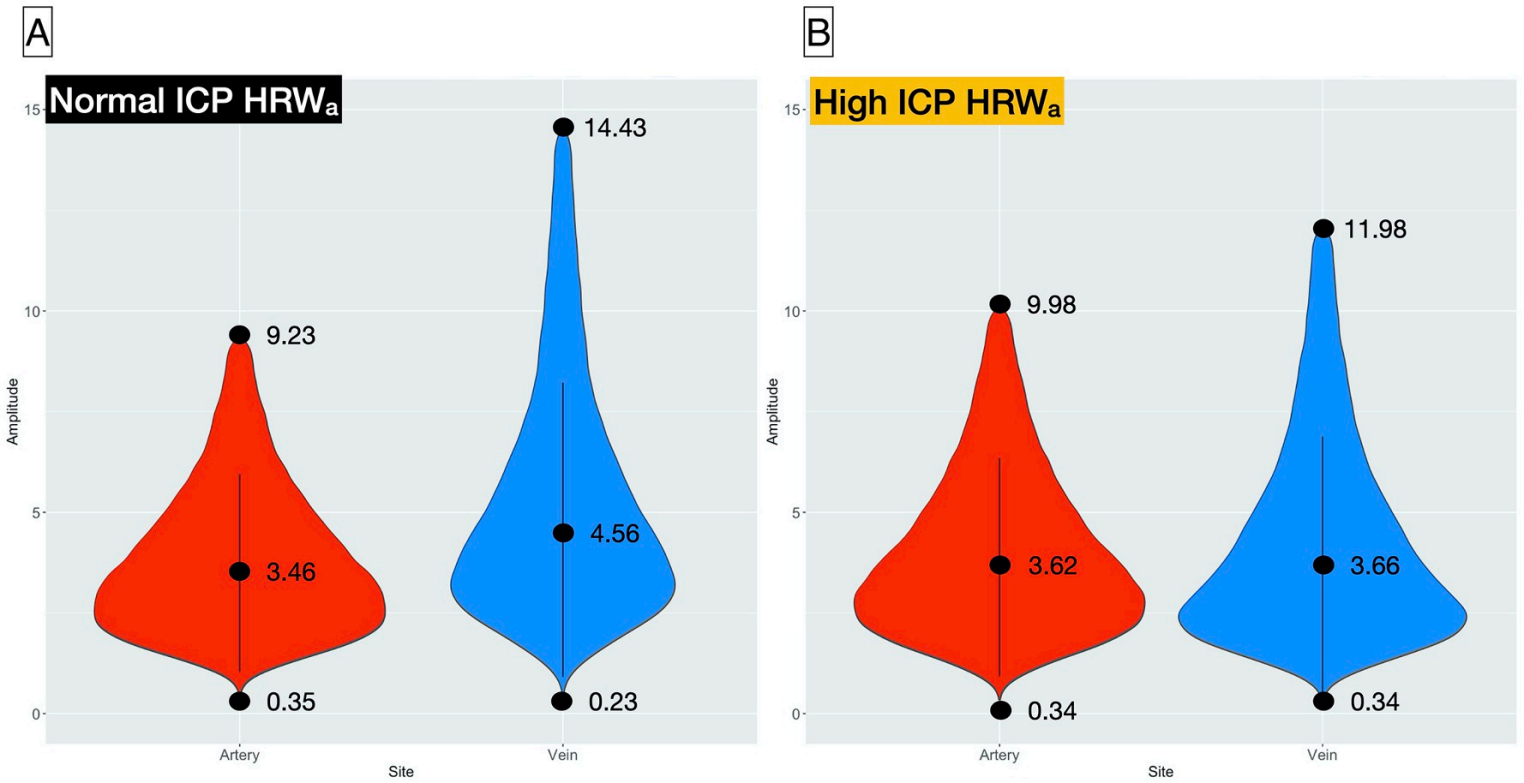
Violin plot of the distribution of the HRW_a_. A) Study group with normal intracranial pressure ICP_n_ (ICP≤25cm water) and B) high intracranial pressure ICP_h_ (ICP>25cm water). The median ± IQR (line and center dot) and range (end dots) are highlighted for each group.

The median venous HRW_a_ was higher in the ICP_n_ group (4.563, range = 0.228–14.434, IQR = 3.656) compared to the ICP_h_ group (3.655, range = 0.339–11.983, IQR = 3.223), this was in contrast to the arterial pulsation amplitude where the median arterial HRW_a_ was higher in the ICP_h_ group (3.616, IQR = 2.715, range = 0.338–9.983) compared to the ICP_n_ group (3.475, IQR = 2.458, range = 0.353–9.233). Between (ICP_h_ and ICP_n_) and within (artery and vein) group differences in the median pulsation amplitudes achieved statistical significance (p<0.0001) for all, except the venous a_n1_ coefficient between groups and the b_n1,2_ within in the ICP_h_ group (Table 2). Therefore there is a reduction in the venous and an increase in arterial pulsation in the ICP_h_ group (Fig 2). Yeo-Johnson transformation (YJ_t_) was undertaken to normalize the HRW_a_ and the Fourier coefficients. As in the non-transformed Fourier terms, statistical significance in the differences between the mean HRW_a_ was sustained between the venous (1.502 and 1.368) and arterial (1.435 and 1.466)(p<0.001) of the ICP_n_ and ICP_h_ groups respectively. Between-group differences were statistically significant in all (p<0.001) except the arterial a_n2_ coefficient. Both within and between-group heterogeneity of the variances was demonstrated using the Levene test (p<0.0001) for the transformed and non-transformed Fourier terms.

**Table 2:** Descriptive statistics.

Parameter	Site	Median	IQR	Min	Max	Range
**ICP_n_**						
**HRWa**	Artery	3.475	2.458	0.353	9.233	8.88
**a_n1_**	Artery	0.078	1.469	-3.924	4.446	8.37
**b_n1_**	Artery	-1.001	1.153	-4.61	4.088	8.699
**a_n2_**	Artery	0.011	0.574	-2.505	2.755	5.26
**b_n2_**	Artery	0.083	0.561	-3.388	3.258	6.647
**HRWa**	Vein	4.563	3.656	0.228	14.434	14.207
**a_n1_**	Vein	0.440^b^	2.057	-6.405	7.045	13.45
**b_n1_**	Vein	-1.324	1.596	-7.211	5.573	12.785
**a_n2_**	Vein	-0.072	0.685	-4.335	4.248	8.583
**b_n2_**	Vein	0.130	0.623	-3.741	4.064	7.805
**ICP_h_**						
**HRWa**	Artery	3.616	2.715	0.338	9.983	9.645
**a_n1_**	Artery	0.179	1.666	-4.826	4.749	9.576
**b_n1_**	Artery	-1.029^w^	1.120	-4.937	4.37	9.307
**a_n2_**	Artery	-0.015	0.605	-2.911	3.158	6.069
**b_n2_**	Artery	0.063^w^	0.622	-3.022	3.256	6.277
**HRWa**	Vein	3.655	3.223	0.339	11.983	11.645
**a_n1_**	Vein	0.364^b^	1.711	-5.707	5.963	11.67
**b_n1_**	Vein	-1.018^w^	1.198	-5.918	5.769	11.687
**a_n2_**	Vein	-0.016	0.613	-3.797	3.731	7.528
**b_n2_**	Vein	0.061^w^	0.597	-3.723	4.246	7.969

Descriptive statistics of the harmonic regression wave amplitude, the cosine and sine coefficients of the first and second harmonics of the two study groups. HRW_a_ = harmonic regression wave amplitude, a_n1,2_ = Fourier cosine coefficient of the first and second harmonic, b_n1,2_ = Fourier sine coefficient of the first and second harmonic, ICP_n_ = normal intracranial pressure group, ICP_h_ = high intracranial pressure group, IQR = interquartile range, Highlighted cells^w^ = within group, Highlighted cells^b^ = between group median differences failed to achieve statistical significance at a level p<0.05.

### Mixed effects linear regression model

**Fig 3:**
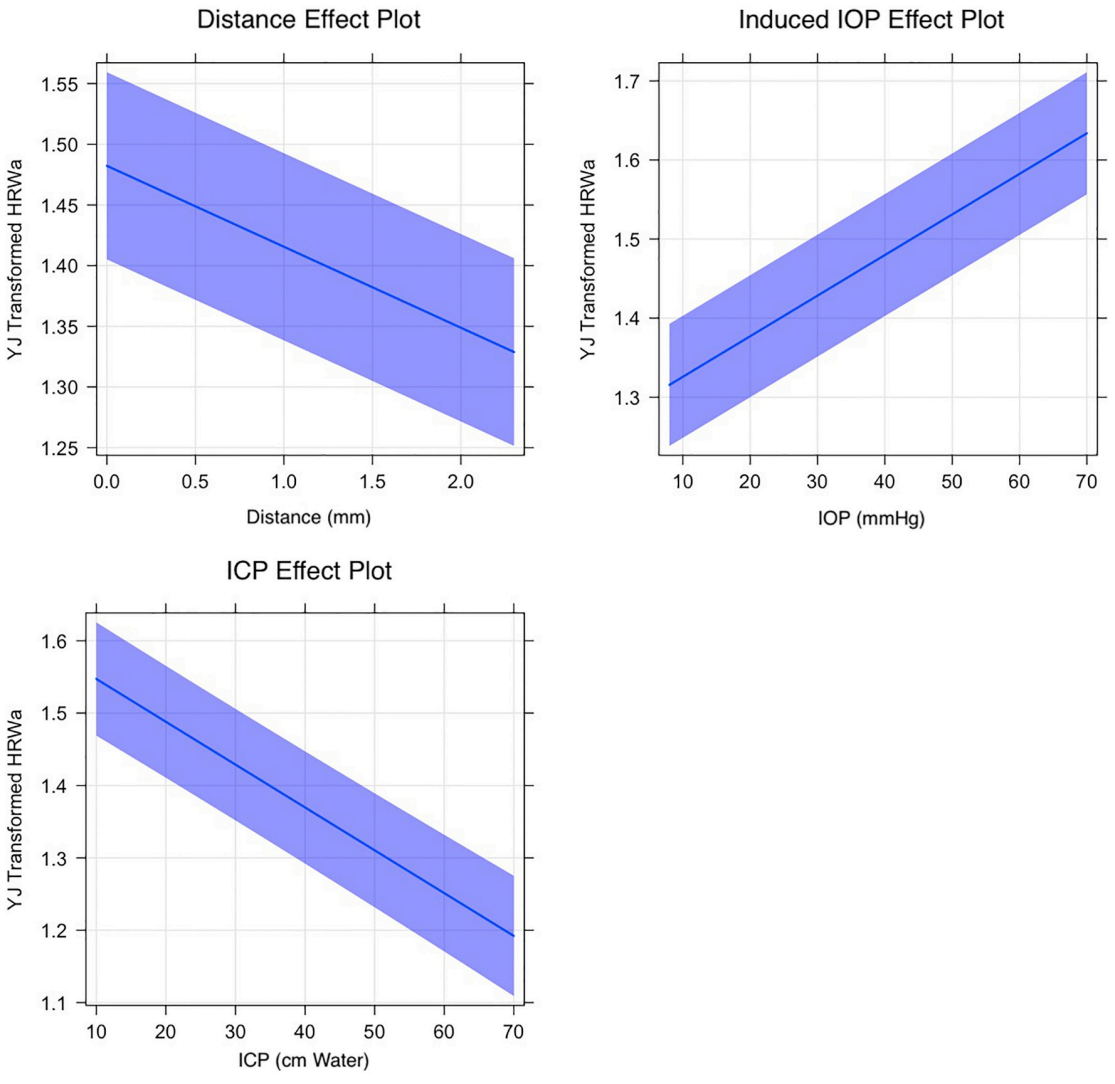
Effect plot of the retinal venous harmonic regression waveform amplitude (HRW_a_). The slope of regression line correlating the HRW_a_ with V_Dist_ was -0.071±0.002 whereas that correlating with ICP was -0.0059±0.0004. There was amplification of the HRW_a_ as indicated by a positive slope of regression with IOP_i_, was 0.0051±0.00006 for all coefficients p-value<0.00001.

**Fig 4:**
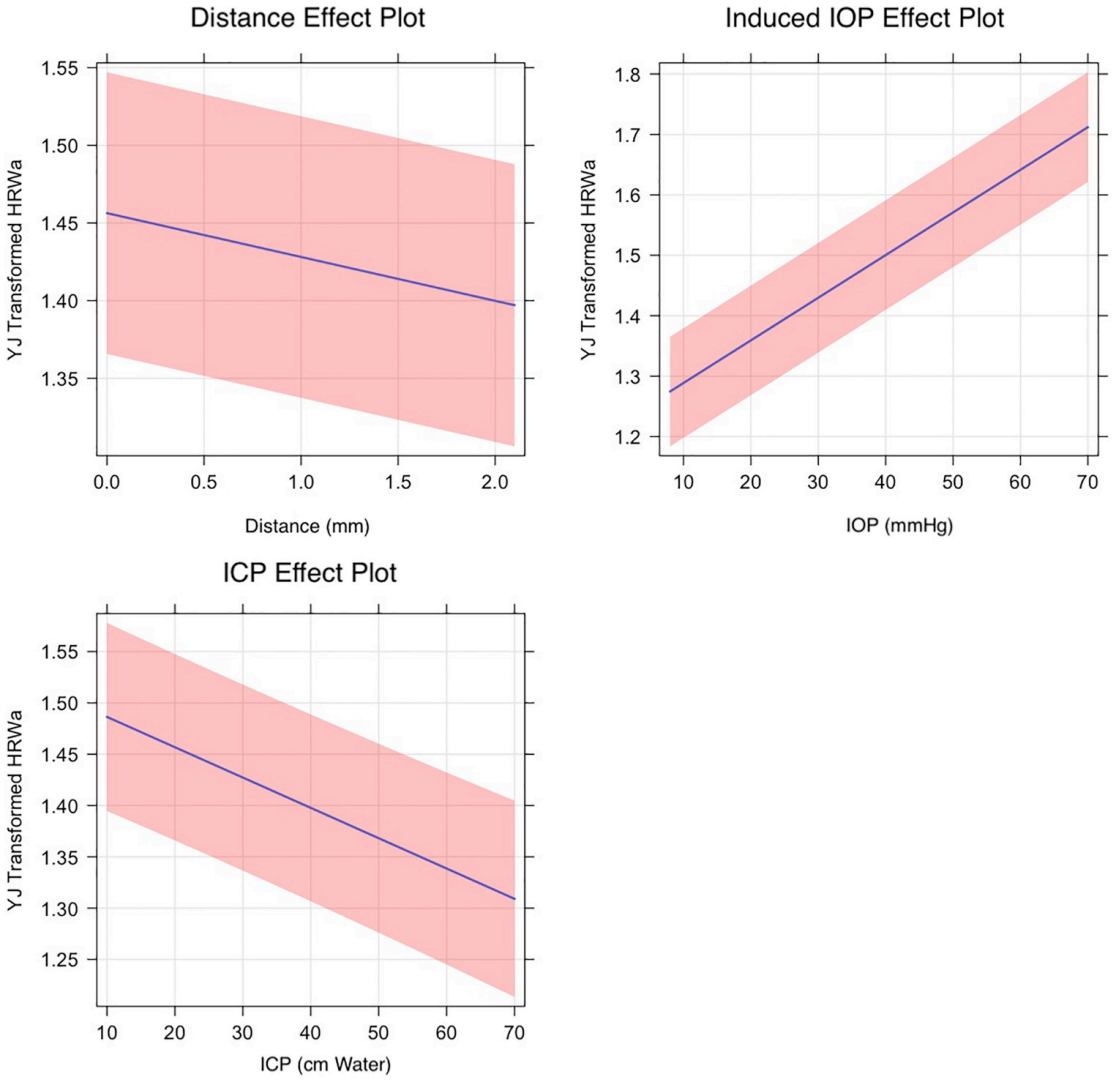
Effect plot of the retinal arterial harmonic regression waveform amplitude (HRW_a_). The slope of regression line correlating the HRW_a_ with V_Dist_ was -0.028±0.0021 whereas that correlating with ICP was -0.0031±0.0004. There was amplification of the HRW_a_ as indicated by a positive slope of regression with IOP_i_, was 0.0071±0.000065 for all coefficients p-value<0.00001.

Correlations between the predictors of the hierarchical multivariate linear regression were undertaken in the YJ_t_ transformation space. In a mixed-effects linear regression model correlating the amplitude of the terms of the Fourier equation with three predictors distance along the vessel (V_Dist_) measured in millimeters (mm) from the center of the optic disc, intracranial pressure (ICP) measured in centimeters water (cm water) and induced intraocular pressure (IOP_i_) measured in millimeters mercury (mmHg). There was a decay in the venous Yeo-Johnson transformed HRW_a_ with both predictors V_Dist_ and ICP. The slope of regression line correlating the HRW_a_ with V_Dist_ was -0.071±0.002 (p-value<0.00001) whereas that correlating with ICP was -0.0059±0.0004 (p-value<0.00001). There was amplification of the HRW_a_ as indicated by a positive slope of regression with IOP_i_, was 0.0051±0.00006 (p-value<0.00001). The corresponding values for the retinal arterial system were -0.028±0.0021, -0.0031±0.0004 and 0.0071±0.000065 (p-value<0.00001) respectively (Figs 3 and 4) in both plots there is attenuation of the HRW_a_ in the retinal venous system with increased distance along the vessel (V_Dist_) and with increasing intracranial pressure (ICP). Amplification of the HRW_a_ with an increase in the induced intraocular pressure (IOP_i_) is noted.

The equations of the regression lines for both the venous HRW_a-YJtV_, and arterial HRW_a-YJtA_ can be derived:

$$\begin{array}{c} HRW_{a-YJtV}=-0.0667\cdot V_{\text{Dist}}\left(\pm 0.002\right)+1.4823\left(\pm 0.04\right)\\ HRW_{a-YJtV}=0.0051\cdot IOP_{i}\left(\pm 0.00006\right)+1.3156\left(\pm 0.04\right)\\ HRW_{a-YJtV}=-0.0059\cdot ICP\left(\pm 0.0004\right)+1.5472\left(\pm 0.04\right) \end{array}$$ (1)

$$\begin{array}{c} HRW_{a-YJtA}=-0.0282\cdot V_{\text{Dist}}\left(\pm 0.0021\right)+1.4564\left(\pm 0.05\right)\\ HRW_{a-YJtA}=0.0071\cdot IOP_{i}\left(\pm 0.00007\right)+1.2746\left(\pm 0.05\right)\\ HRW_{a-YJtA}=-0.0031\cdot ICP\left(\pm 0.0004\right)+1.4864\left(\pm 0.05\right) \end{array}$$ (2)

Where the coefficients of all equations were highly statistically significant (p<0.00001). For both retinal vascular systems, the negative coefficients of V_Dist_ and ICP indicate that decay in retinal venous pulsation amplitude in response to these predictors and a positive correlation with IOP_i_. From Eqs (1) and (2), it can be noted that the retinal vascular pulse wave attenuation of the retinal veins is approximately two times that of the retinal arteries.

**Fig 5:**
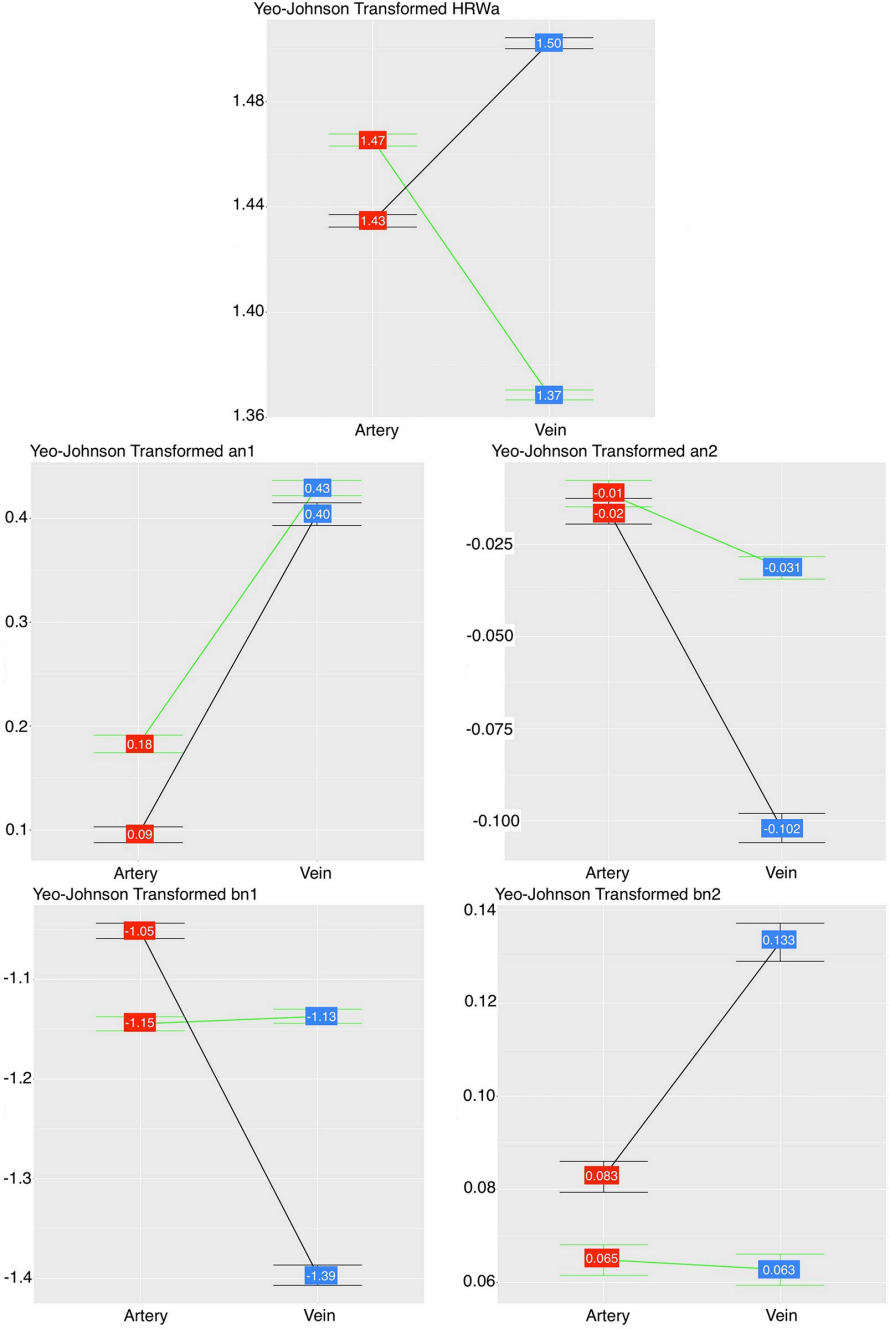
Interaction plot of the harmonic regression waveform amplitude (HRW_a_), cosine (a_n1,2_) and sine (b_n1,2_) coefficients of the first and second harmonics. In both the high (h) and normal (n) intracranial pressure groups for both vascular systems there was interaction of the HRW_a_ and b_n1_, no interaction of the a_n1_ term and ordinal interaction of the a_n2_ and b_n2_ terms. Therefore the sine coefficient (b_n1_) and the second harmonic coefficients reflects the retinal vascular pulse response to ICP and the cosine coefficient (a_n1_) follows the IOP pulse wave response on the vessel wall. Color code: green = ICP_h_, black = ICP_n_ study group.

A graphical display of the interactions is demonstrated in Fig 5. They display the relationship between the dependent variable on the y-axis i.e. mean pulsation amplitude (HRW_a-YJt_ and Fourier coefficients, which are the cosine (a_n_) and sine (b_n_)) and the independent variable on the x-axis i.e. vessel type (artery and vein), also called the main effect. This relationship is evaluated under different ICP levels grouped into two factor levels (high and normal). It is worth considering the specific terminology used when describing interaction plots, these pertain to two observations reported from these plots. The first if there is a main effect, this is present when the line connecting the mean pulsation amplitudes between two different vessel types is sloped, which indicates the difference in the mean pulsation amplitude between is statistically significant. The steeper the slope of the line, the greater the magnitude of the main effect. In contrast, a main effect is absent when the line connecting the mean pulsation amplitudes is horizontal and therefore parallel to the x-axis, in this state, there is no statistically significant difference in the mean pulsation amplitude between the artery and vein. The second observation is the presence of an interaction with the grouping factor (ICP), this may be disordinal when there is an intersection of the two lines connecting the means, or ordinal when the lines are non-parallel and non-intersecting [49].

From Fig 5, there is a significant main effect as a result of a statistically significant difference between the mean HRW_a_ pulsation amplitudes between the vessels. Also, there is disordinal (cross-over) interaction between the two ICP groups. Noted are a high mean arterial and low mean venous HRW_a-YJt_ pulsation amplitude in the ICP_h_ group and conversely for the ICP_n_ group. The a_n1_ showed no interaction between the ICP groups, however, there was a significant main effect. Based on a lack of interaction with ICP, the coefficient follows the effect of the IOP wave, and therefore the effects of IOP_i_ on the retinal vessel wall. The a_n2_ showed ordinal (non-intersecting, non-parallel) interaction where between-group differences in the value of this coefficient existed only in the retinal veins (Fig 5). The interaction plots the sine (b_n1_) coefficient show a strong interaction between ICP groups and the loss of main effects in the ICP_h_ group as indicated by the parallel line joining the mean pulsation values to the x-axis. The sine (b_n2_) coefficient showed ordinal interaction with ICP but there was a loss of a main effect in the ICP_h_ group. Based on these observations the b_n1_ and the second harmonic coefficients mirror the retinal vascular response to the ICP wave.

**Fig 6:**
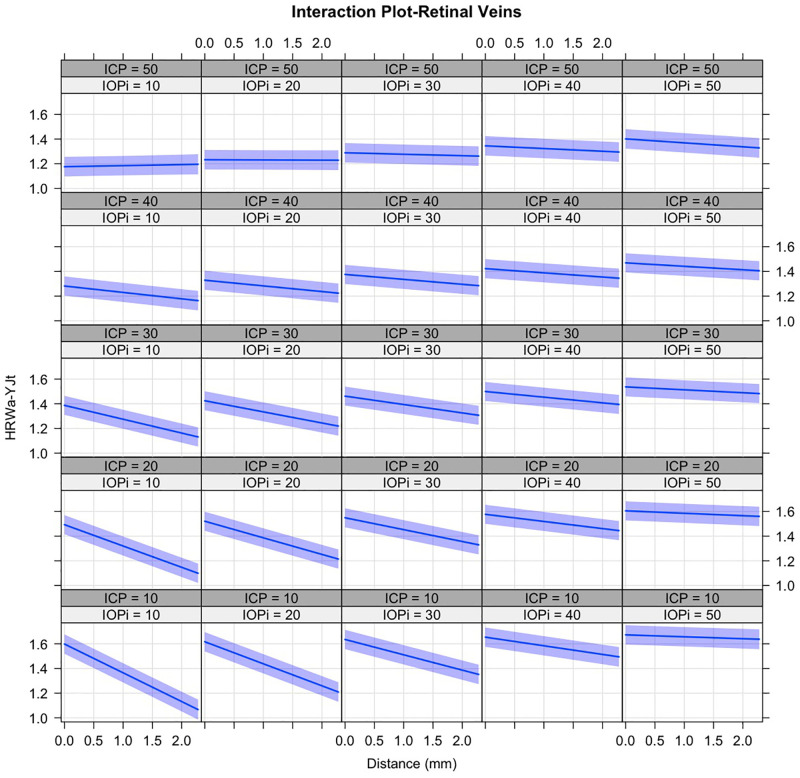
Trellis graph demonstrating the mixed effects linear regression model with interactions of the Yeo-Johnson transformed venous harmonic regression waveform amplitude (HRW_a-YJt_). Distance is measured in millimeters (mm) from the center of the optic disc. The 95% confidence intervals are shown around the regression lines. Noted is a reduction of the slope of the regression with increased ICP (columns) and IOP_i_ (rows). Whereas the y-intercepts of the regression lines are reduced with increased ICP, they are increased with higher IOP_i_. The progressively lower y-intercepts of the regression lines along the lower left to upper right diagonal indicate that a higher IOP_i_ is required to induce a venous pulsation with higher ICP, which will be of a lower amplitude.

**Fig 7:**
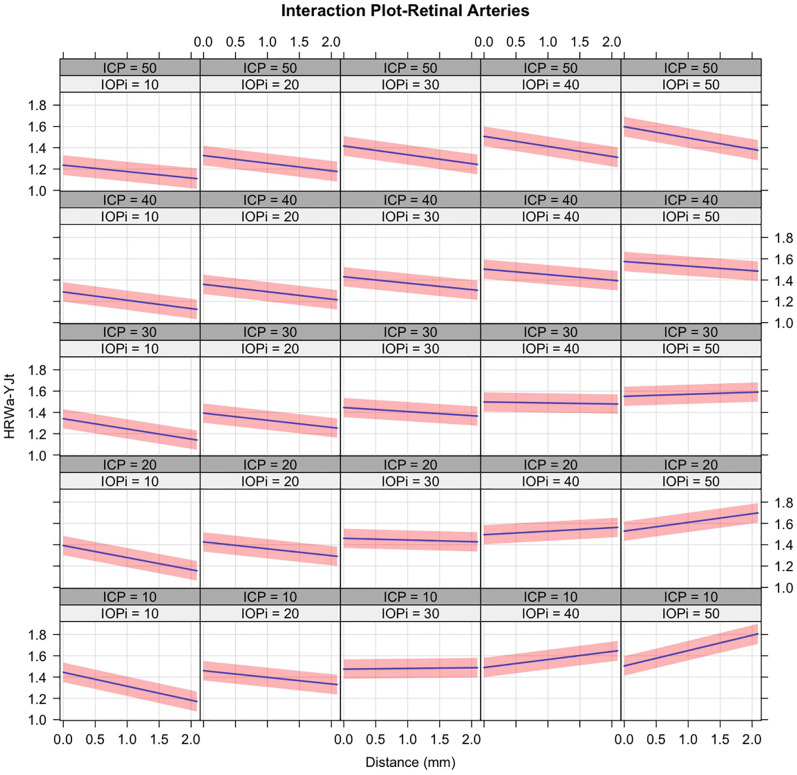
Trellis graph demonstrating the mixed effects linear regression model with interactions of the Yeo-Johnson transformed arterial harmonic regression waveform amplitude (HRW_a-YJt_). Distance is measured in millimeters (mm) from the center of the optic disc. The 95% confidence intervals are shown around the regression lines. Noted is a increase of the slope of the regression with increased ICP (columns) and IOP_i_ (rows) particularly with ICP in the pathological range. Contrary to the retinal veins the progressively higher y-intercepts of the regression lines along the lower left to upper right diagonal indicate that amplification of the retinal arterial pulsation occurs with a higher ICP. A reversal of the slope of the regression at ICP 10–20 at an IOPi >30 mmHg is seen, this is likely a spurious result as a consequence of the boundary conditions of the mixed effects linear model.

A mixed-effects linear regression hierarchal model was used to quantify the interactions between the predictors for the HRW_a-YJt_, for the retinal veins (Fig 6) and arteries (Fig 7). Where HRW_a-YJt_ is the Yeo-Johnson transformed value of the retinal vascular pulse, ICP is in cm water, IOP_i_ is the induced IOP in mmHg, and V_Dist_ is in mm from the center of the optic disc:

$$\begin{array}{c} HRW_{a-YJtV}=-0.3638\left(\pm 0.013\right)\cdot V_{\text{Dist}}+0.00091\left(\pm 0.00029\right)\cdot {IOP}_{i}\\ -0.01149\left(\pm 0.00052\right)\cdot ICP+0.0071\left(\pm 0.00036\right)\cdot V_{\text{Dist}}\cdot {IOP}_{i}\\ +0.0085\left(\pm 0.00041\right)\cdot V_{\text{Dist}}\cdot ICP+0.000094\left(\pm 0.0000093\right)\cdot {IOP}_{i}\cdot ICP\\ -0.0001612\left(\pm 0.000012\right)V_{\text{Dist}}\cdot {IOP}_{i}\cdot ICP+1.69 \end{array}$$ (3)

$$\begin{array}{c} HRW_{a-YJtA}=-0.2374\left(\pm 0.017\right)\cdot V_{\text{Dist}}+-0.00038\left(\pm 0.00038\right)\cdot {IOP}_{i}\\ -0.00708\left(\pm 0.00057\right)\cdot ICP+0.0089\left(\pm 0.00053\right)\cdot V_{\text{Dist}}\cdot {IOP}_{i}\\ +0.00376\left(\pm 0.00059\right)\cdot V_{\text{Dist}}\cdot ICP+0.000188\left(\pm 0.000013\right)\cdot {IOP}_{i}\cdot ICP\\ -0.0002\left(\pm 0.000018\right)V_{\text{Dist}}\cdot {IOP}_{i}\cdot ICP+1.50 \end{array}$$ (4)

Both equations can be rearranged in terms of V_Dist_, which allows some interaction terms to be eliminated:

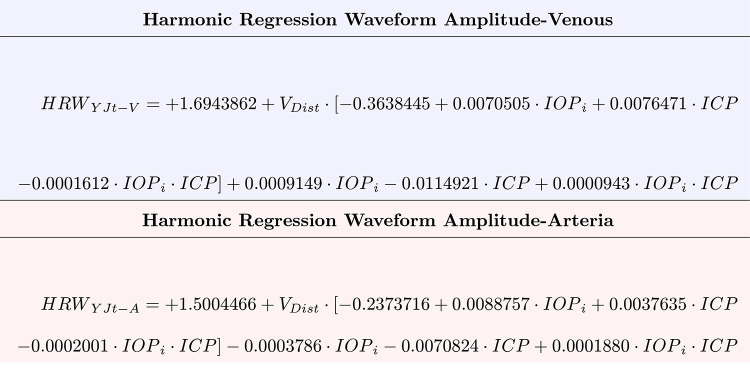


The interactions are displayed graphically in Figs 6 and 7.

All coefficients of the regression equations with interactions achieved statistical significance (p<0.001), except the arterial IOP_i_ coefficient (p = 0.32). The overall interaction model predicting the HRW_a-YJtV_ had a total explanatory power (conditional R^2^) of 38.7%, in which the fixed effects explained 8.8% of the variance (marginal R^2^). Within this model the effect of V_Dist_ and ICP were significant ($\beta_{V_{\text{Dist}}}=-0.42$, se = ±0.015, p < 0.00001) and (*β*_*ICP*_ = -0.42, se = ±0.019, p < 0.00001). Whereas that for IOP_i_ was small ($\beta_{{IOP}_{\text{i}}}=0.031$, se = ±0.0097, p < 0.00001).

It can be observed from Fig 6 that there is a reduction of the slope of the regression line with increased ICP (columns) and IOP_i_ (rows). Whereas the intercept of the regression lines are reduced with increased ICP, observed as a progressive reduction in venous pulsation, the converse occurs with raised IOP_i_. Following a diagonal of the trellis graph (Fig 6) from the lower left to the upper right, where both ICP and IOP_i_ increase, it can be observed that a higher IOP_i_ is required to induce venous pulsations of progressively lower amplitude in the presence of a higher ICP. The interactions in the retinal arteries seen in (Fig 7) depend on whether ICP is pathologic or physiologic. Where ICP is pathological, the intercept of the regression lines are is increased with increased IOP_i_. At the physiologic ICP range, there is an increase in the intercept therefore an increase in retinal arterial pulse amplitude with increasing IOP_i_, however, this increase in intercept is less than that in the pathological ICP range. A reversal of the slope of the regression at ICP 10–20 at an IOPi >30 mmHg is seen. This is likely a spurious result as a consequence of the boundary conditions of the mixed-effects linear model. Contrary to the retinal veins when a diagonal of the trellis graph (Fig 7), followed from the lower left to the upper right, where both ICP and IOP_i_ increase, a corresponding increase in retinal arterial pulse amplitude can be observed.

The overall interaction model predicting the HRW_a-YJtA_ has a total explanatory power (conditional R^2^) of 42%, in which the fixed effects explained 5.8% of the variance (marginal R^2^). Within this model the effect of V_Dist_ and ICP were significant ($\beta_{V_{\text{Dist}}}=-0.26$, se = ±0.019, p < 0.00001) and (*β*_*ICP*_ = -0.21, se = ±0.017, p < 0.00001). Whereas that for IOP_i_ was small and did not achieve statistical significance ($\beta_{{IOP}_{\text{i}}}=-0.013$, se = ±0.013, p < 0.32).

The statistical significance of the coefficients of the linear regression model with interactions of the HRW_a-YJt_ and the terms of the Fourier series are summarised in Table 3.

**Table 3:** Estimated interaction model coefficients.

	HRW_a_	a_n1_	b_n1_	a_n2_	b_n2_
**Artery**					
V_Dist_	-0.237±0.017	-0.193±0.05171	0.3681±0.0551	0.2315±0.028	-0.1451±0.0303
IOP_i_	-0.00038±0.00038*	0.0084±0.00113	-0.01155±0.0012	-0.00228±0.00061	-0.0047±0.00066
ICP	-0.0071±0.00057	-0.0073±0.0017	0.02934±0.0018	0.0069±0.00091	-0.0248±0.00099
V_Dist_ ⋅ IOP_i_	0.0089±0.00053	-0.0155±0.0016	-0.0042±0.00167	0.00051±0.00084*	0.0041±0.00091
V_Dist_ ⋅ ICP	0.0037635±0.00059	0.00221±0.0018*	-0.0049±0.0019	-0.00624±0.00095	0.00094±0.001035*
IOP_i_ ⋅ ICP	0.0001880±0.000013	0.00013±0.000039	-0.000156±0.000041	0.000088±0.000021	0.00015±0.000023
V_Dist_ ⋅ IOP_i_ ⋅ ICP	-0.0002±0.000018	0.000267±0.000053	0.000124±0.000056	0.00001±0.000029*	-0.000013±0.000031*
**Vein**					
V_Dist_	-0.3638±0.013	-0.0847±0.051*	-0.5504±0.0541	-0.0431±0.0242*	-0.1225±0.0254
IOP_i_	0.00091±0.00029	0.0232±0.0011	-0.048±0.0012	-0.0032±0.00054	-0.000195±0.00056*
ICP	-0.0115±0.00052	0.0181±0.0020	0.0031±0.0022*	0.01795±0.00097	-0.00682±0.001
V_Dist_ ⋅ IOP_i_	0.0071±0.00036	-0.0083±0.0014	0.0395±0.0015	0.00156±0.00067	0.001±0.0007*
V_Dist_ ⋅ ICP	0.0076±0.00041	-0.0026±0.0016*	0.0271±0.00173	-0.00092±0.00077*	-0.00184±0.00081
IOP_i_ ⋅ ICP	0.000094±0.0000093	-0.00023±0.000036	0.0011±0.00004	-0.000013±0.000018*	-0.00012±0.000018
V_Dist_ ⋅ IOP_i_ ⋅ ICP	-0.00016±0.000011	0.00019±0.000041	-0.0011±0.000045	0.000023±0.00002*	0.000088±0.000021

Estimated interaction model coefficients of the terms of the first two harmonics of the Fourier series. CS = computationally singular. Highlighted cells (*) represent coefficients that failed to achieve statistical significance. p-value<0.001 for the rest of the coefficients.

## Discussion

We quantified the difference between retinal venous and arterial pulse amplitudes in subjects with high and normal intracranial pressures over a range of induced intraocular pressures, which demonstrated a reduction in retinal venous and an increase in arterial pulsation amplitudes with raised ICP. In our current study both the fixed effects and the random factors in the linear models accounted for approximately 40% and the predictors accounted for less than 10% of the variance in the study population, despite the linear models’ coefficients achieving a high level of statistical significance the variance between individuals (heteroscedasticity) in the vascular pulsation amplitudes precludes the use of the model as a predictor of ICP. However, the interpretive nature of the linear models allowed further analysis of the retinal vascular responses to a range of ICP values.

One of the significant findings was the reduction in the median venous and the increase in the median arterial HRW_a_ in the ICP_h_ group (Fig 2). Spontaneous venous pulsation (SVP) is a result of the pressure gradient along the retinal vein as it traverses the lamina cribrosa. Loss of spontaneous venous pulsation is a recognized sign of increased ICP [50, 51], as increase in ICP increases intracranial pulse pressure. Consequently, SVP ceases when the ICP pulse pressure is equal to intraocular pulse pressure [52]. Although SVPs are classically described as a categorical and binary clinical sign, in this work and previous reports we demonstrated that modified photoplethysmography can provide a quantitative measure of this clinical sign non-invasively [29, 50]. Whereas changes in the retinal venous system with high ICP have been described in the literature, where a reduction in retinal venous compliance occurs from the combined influence of increased intravenous pressure, venous distension, and increase in venous wall tension [5, 7, 53, 54]. The literature has been less definite on the changes in the retinal arterial circulation, Querfurth and Mitrra et al. reported a reduction in retinal arterial flow velocities in mild to moderate raised ICP [55]. Querfurth found a paradoxical increase in central retinal and the ophthalmic arterial flow velocities with an ICP ≥450 mm water [56]. Other sonographic studies reported an increase in pulsatility index of the central retinal artery regardless of ICP levels [2, 57]. Interpretations of the vascular response remain hypothetical. Modified Photoplethysmography cannot inform why differences in pulsation patterns exist between the vessels in the two study groups.

Differences between the retinal arterial and venous pulsation amplitude, patterns, and attenuation distance arise from unique properties of the pressure-flow wave, resistance, and vessel wall structural characteristics. Consequently, a range of reported prevalences of detectable retinal vascular pulsation is dependent on the measurement methodology. Clinically observable spontaneous retinal venous pulsations are reported in 67% to 98% of normal eyes [19, 51, 58, 59], compared to the arterial pulse where spontaneous pulsations have been reported to be physiologically absent and only associated with vascular pathology [60–62]. As an extension of clinical observation, modern imaging technology has facilitated the detection and measurement of retinal vascular pulsations and has rendered the arterial pulse measurable physiologically. Clinically available methods include: dynamic fluorescein [63] and indocyanine angiography [64, 65], near-infrared reflectance ophthalmoscopy [59], swept-source optical coherence tomography [66], adaptive optics imaging [67], and the Dynamic Vessel Analyzer [68]. In the largest study to date, McHugh et al. reported the detectability of spontaneous retinal venous pulsation in a normal population using near-infra-red videography. Their cross-sectional study design was conducted on 105 patients with presumed normal intracranial pressure, spontaneous venous pulsation was detected in 97% to 100% of subjects [69]. Whereas this study investigated the detectability of retinal venous pulsation, the prevalence of retinal arterial pulsations was not reported. Using the same technology, Moret et al., used principal component analysis to filter videos of the optic nerve and temporal peripapillary area. They reported a low detection rate as they observed spontaneous venous pulsation in 5 of 10 (50%) subjects. This was likely due to the low device frame rate of 9 frames per second used in their study. However, they reported various arterial pulsation patterns including the serpentine movement of the principal arteries, pulsatile vessels of the optic nerve head, vessel displacement, and arterio-venous mechanical coupling [59]. Modified Photoplethysmography, distinct from other technologies, quantifies the retinal vascular pulse amplitude in the Fourier domain. This method has been validated in previous work [29, 31, 43, 50] where we reported quantifiable differences in the normal retinal vessel pulsations. In a study on 28 eyes of 16 normal subjects, we reported the normal median venous pulse amplitude was approximately 1.5 times and the rate of decay was twice that of the arterial pulse wave [29]. Similarly, in the current study the venous pulse amplitude was 1.3 times and the coefficient of decay 2.3 times that of the arterial. Therefore physiological properties account for differences in pulse amplitude differences between the arteries and the veins. From the weighted *β* coefficients of linear models with interactions (Eqs (3) and (4)) it is noted that both V_Dist_ and ICP have a similar damping effect on venous wall ($\beta_{V_{\text{Dist}}}=-0.42$, se = ±0.015, *β*_*ICP*_ = -0.42, se = ±0.019, p < 0.00001). As described above, reduction in venous wall compliance as a result of venous distension accounts for this effect, the damping effect of ICP on the arterial wall is less than V_Dist_ ($\beta_{V_{\text{Dist}}}=-0.26$, se = ±0.019, *β*_*ICP*_ = -0.21, se = ±0.017, p < 0.00001), both structural differences of different vessel types may account for differences in pulsation decay rates.

In previous work, we quantified the decay in the retinal vascular pulse amplitude from a population of healthy subjects and reported that although the amplitude of the retinal venous pulse is higher it decays over a shorter distance compared to that of the arterial [29]. Differences in attenuation and amplification in retinal vascular pulse between the retinal arteries and veins in response to V_Dist_, ICP and IOP_i_ (Figs 3 and 4) are due to variations in retinal vascular compliance and resistance. Whereas systemic arterial compliance plots are curvilinear, this relationship is sigmoidal in the veins, resulting in a 19 to 24 times difference in systemic venous compliance for an equivalent transmural pressure and cross-sectional area [70–75]. This relationship has not been quantified directly in the retinal vascular system, however, among the many structural differences, including differences in collagen content, the thinner venous wall consists of a muscle layer that is 3–4 layers thick compared to the 5–7 layers found in the artery [76] indicates the lower compliance in this part of the vascular tree. Resistance to hemodynamic flow is set by circulating blood, net length, branching pattern, and cross-sectional area of the specific circulation. Similar to the systemic circulation, the primary site of resistance resides in the small arteries and arterioles. However, unique to the ocular circulation, the retinal vein, which is the low resistance part of the retinal circulation, is subject to an external compression force in the form of intraocular pressure, thereby acting as a Starling resistor, which is defined as a mechanism that maintains constant flow through a collapsible tube when the latter is surrounded by varying pressure and contained in a pressurized chamber, the mechanisms and significance of which remains unknown [77–79]. Theoretically, both compliance and resistance are dependent on tension (T) in the vessel wall, which according to Laplace’s law [80], is proportional to the product of the blood vessel radius (r) and transmural pressure (P_tm_, defined as the difference between intra-luminal and extra-luminal pressures)

$$\begin{array}{c} T=P_{tm}\cdot r \end{array}$$

For pulsations to occur in a vessel segment, either a pressure gradient must exist or the transmural pressure must be reduced to overcome this mural tension. Both IOP_i_ and ICP influence the mural tension. The retinal veins are more susceptible than arteries to changes in IOP_i_ and ICP due to the thinner walls, wider radius, lower intraluminal pressure, and hence lower resistance in the veins compared to the arteries. There are two mechanisms by which ICP and IOP_i_ can influence wall tension and therefore the retinal vascular pulse:

1. Reduction in transmural pressure: The lower limit for retinal venous pressure is determined by the ocular perfusion pressure, which is the difference between mean arterial pressure and IOP_i_, therefore the central retinal venous pressure must exceed IOP_i_ to prevent venous wall collapse [81]. The lower limit in the retinal arterial pressure is the diastolic pressure [82, 83]. As IOP_i_ rises, transmural pressure decreases, resulting in an increase in vessel wall compliance through lowering wall tension. The vessel collapses partially or completely during a period of the cardiac cycle when the transmural pressure is between diastolic and systolic intra-luminal retinal vascular pressure (within the pulse pressure range). As systolic pressure exceeds the transmural pressure, the vessel re-fills, producing vascular pulsations. [60, 84]. When transmural pressure is negative, the vessel wall collapses, and pulsations in the vascular segment cease. Because the collapse pressure in the retinal veins is lower than in the retinal arteries, a higher the transmural pressure range (and hence higher IOP_i_) influences the thicker-walled arteries after venous wall collapse occurs, which may explain the different IOP_i_ ranges to induce pulsations in the retinal veins and arteries.
2. Reduction in retinal venous compliance: The effect of raised ICP on the retinal circulation is to increase resistance in the venous circulation [62] combined with reduced venous compliance due to vessel wall distension [71, 85]. The mechanism for increased retinal arterial pulsations, in this case, remain unclear. However, analogous to the observation of arteriolar pulsations reported in type A central retinal vein occlusion characterized by pulsatile arterial filling [62], possible mechanisms include:
  1. Elevated IOP_i_ above central retinal arterial diastolic pressure as hypothesized with pulsations of the central retinal artery occurring due to ophthalmodynamometry [82, 83]. Together with reduced venous compliance of the artery wall, may limit the damping of the vascular pulse pressure, resulting in increased arterial pulsations proportional to elevated ICP [86].
  2. Augmentation of the vascular pressure wave through wave reflections and possibly widening of the retinal vascular pulse pressure. Although this mechanism has not been demonstrated in the retinal vessels, wave reflections are ubiquitous throughout the circulation, particularly at sites of higher input impedance. Impedance is a complex value frequency domain parameter with both magnitude and phase, it indicates alterations in compliance, resistance, and inertance. Mismatching vascular impedances reduces the pulse transmission efficiency for the propagating pulse towards the venous side of the circulation, consequently wave reflections arise, which augment the pressure wave amplitude and reduce flow due to phase differences [34, 36, 87, 88].

We hypothesize these mechanisms could explain the changes with the pulsation amplitudes in Figs 6 and 7, where ICP and IOP_i_ have different influences on the retinal vascular pulse, primarily through the venous side of the circulation due to the thinner wall, lower resistance, and higher compliance.

We did not evaluate the spectral properties of the IOP nor the ICP wave in our study. However, the interaction plots (Fig 5), which compare the pulsation amplitude between the two study groups, suggest a that the Fourier coefficients convey information from the vessel walls, where the cosine (a_n1_) and sine coefficient (b_n1_) mirror the effects of IOP_i_, and the ICP wave, respectfully. Other investigators reported on the spectral properties of the latter two pressure waves, Evans et al. analyzed the IOP pulse’s higher spectral components up to the fourth harmonic and Bozic et al. compared the spectral components of the IOP pulse wave up to the fifth harmonic, however, the hemodynamic significance of the Fourier analysis remains obscure [89–91]. Takizawa et al., in an experimental study, described the frequency spectrum and amplitude transfer function at different locations in the craniospinal axis, they concluded that the ICP pulse waveform is almost the same at any location in the intracranial space and the origin of the ICP pulse wave is the pulsation of the brain parenchyma derived from cerebral arterial blood flow [92]. None of these studies specifically identified Fourier coefficients associated with these pressure waves, our results may guide future predictive modeling for non-invasive estimation of ICP. The accessibility of the retinal vasculature makes it a favorable means of non-invasive prediction of ICP. Additionally, a particularly strong association between retinal and intracranial pathology arises from the common embryonic origin shared between the retina and the brain, consequently, both animal and experimental evidence demonstrate similarities in circulatory physiology [93]. During embryonic development, the retina and optic nerve extend from the diencephalon, and are thus considered part of the central nervous system [94]. They possess similar structural and functional features such as autoregulation, a blood-retina barrier, and glial cell connections [94–97]. Non-invasive ICP prediction would not only reduce the risks of invasive procedures, but it would provide an accessible avenue to explore neuro-ophthalmic, retinal vascular, and glaucomatous optic neuropathy and perhaps provide further insight into the pathogenesis of these disorders.

In summary, the retinal vascular changes are a consequence of a complex interaction between the retinal hemodynamics (retinal pressure-flow wave and vessel wall), IOP, ICP, and systemic blood pressure, in addition to biomechanical properties of the lamina cribrosa [1, 5, 54, 98]. In pathological ICP ranges, whereas the retinal venous pulsation amplitude is reduced due to decreased wall compliance and venous distension, the retinal arteries demonstrate an increase in pulse amplitude. Mechanisms in the arteries remain hypothetical. However, possible mechanisms include a reduction in damping of the arterial pulse pressure, augmentation of the arterial pressure wave through wave reflections, and widening of the retinal arterial pulse pressure. Although both are generated by the cardiac cycle, the influence of IOP and ICP pressure waves on the vessel wall might be evaluated by separate Fourier coefficients.

Limitations of our study include the limited number of subjects and the lack of simultaneous blood pressure measurements, which may have assisted in the interpretation of the findings in the retinal arterial system.

## Conclusion

The retinal vascular pulsation characteristics in the ICP_h_ group showed high retinal arterial and low venous pulsation amplitudes. Interactions between retinal vascular pulsation amplitude and ICP suggest that the Fourier sine coefficient b_n1_ reflects the retinal vascular response to the ICP wave. Although a matrix of regression lines showed high linear characteristics, the low model explanatory power precludes its use as a predictor of ICP. These results may guide future predictive modelling in non-invasive estimation of ICP using modified photoplethysmography.

## Supporting information

S1 Data (CSV)
